# Persistent Oxidative Stress and Inflammasome Activation in CD14^high^CD16^−^ Monocytes From COVID-19 Patients

**DOI:** 10.3389/fimmu.2021.799558

**Published:** 2022-01-14

**Authors:** Silvia Lucena Lage, Eduardo Pinheiro Amaral, Kerry L. Hilligan, Elizabeth Laidlaw, Adam Rupert, Sivaranjani Namasivayan, Joseph Rocco, Frances Galindo, Anela Kellogg, Princy Kumar, Rita Poon, Glenn W. Wortmann, John P. Shannon, Heather D. Hickman, Andrea Lisco, Maura Manion, Alan Sher, Irini Sereti

**Affiliations:** ^1^ HIV Pathogenesis Section, Laboratory of Immunoregulation, National Institute of Allergy and Infectious Diseases, National Institutes of Health, Bethesda, MD, United States; ^2^ Immunobiology Section, Laboratory of Parasitic Diseases, National Institute of Allergy and Infectious Diseases, National Institutes of Health, Bethesda, MD, United States; ^3^ Immune Cell Biology Programme, Malaghan Institute of Medical Research, Wellington, New Zealand; ^4^ AIDS Monitoring Laboratory, Frederick National Laboratory for Cancer Research, Leidos Biomedical Research, Inc., Frederick, MD, United States; ^5^ Clinical Monitoring Research Program Directorate, Frederick National Laboratory for Cancer Research, Leidos Biomedical Research, Inc., Frederick, MD, United States; ^6^ Division of Infectious Diseases and Tropical Medicine, Georgetown University Medical Center, Washington, DC, United States; ^7^ Division of Infectious Diseases and Travel Medicine, MedStar Georgetown University Hospital, Washington, DC, United States; ^8^ Section of Infectious Diseases, MedStar Washington Hospital Center, Washington, DC, United States; ^9^ Viral Immunity and Pathogenesis Unit, Laboratory of Clinical Immunology and Microbiology, National Institutes of Allergy and Infectious Diseases, National Institutes of Health, Bethesda, MD, United States

**Keywords:** COVID-19, NLRP3 inflammasome, oxidative stress, lipid peroxidation, CD14^high^CD16^−^ monocytes

## Abstract

The poor outcome of the coronavirus disease-2019 (COVID-19), caused by SARS-CoV-2, is associated with systemic hyperinflammatory response and immunopathology. Although inflammasome and oxidative stress have independently been implicated in COVID-19, it is poorly understood whether these two pathways cooperatively contribute to disease severity. Herein, we found an enrichment of CD14^high^CD16^−^ monocytes displaying inflammasome activation evidenced by caspase-1/ASC-speck formation in severe COVID-19 patients when compared to mild ones and healthy controls, respectively. Those cells also showed aberrant levels of mitochondrial superoxide and lipid peroxidation, both hallmarks of the oxidative stress response, which strongly correlated with caspase-1 activity. In addition, we found that NLRP3 inflammasome-derived IL-1β secretion by SARS-CoV-2-exposed monocytes *in vitro* was partially dependent on lipid peroxidation. Importantly, altered inflammasome and stress responses persisted after short-term patient recovery. Collectively, our findings suggest oxidative stress/NLRP3 signaling pathway as a potential target for host-directed therapy to mitigate early COVID-19 hyperinflammation and also its long-term outcomes.

## Introduction

Severe acute respiratory syndrome coronavirus 2 (SARS-CoV-2), the causative agent of coronavirus disease 2019 (COVID-19), emerged in late 2019 and rapidly spread worldwide, leading to approximately 5 million deaths to date (1). Although the majority of infected individuals are asymptomatic or experience mild disease, about 20% of patients with COVID-19 develop moderate/severe manifestations mainly characterized by hypoxia and extensive pneumonia (2). Patients occasionally progress to critical condition as a result of pathological complications related to acute respiratory distress syndrome (ARDS), endothelial dysfunction and coagulopathy, leading to multi-organ failure and consequently death (3–6).

An exacerbated inflammatory response has been implicated as a major cause of morbidity and mortality in patients with COVID-19 (7, 8). Overall, elevated levels of circulating inflammatory markers, namely, C-reactive protein (CRP), ferritin, D-dimer and a wide range of inflammatory cytokines/chemokines, namely, the pro-inflammatory cytokines IL-1β and IL-18 have been associated with poor disease outcome (9–11). IL-1β and IL-18 production rely on inflammasome activation upon sensing of pathogen/damage-associated molecular patterns (PAMPs/DAMPs) by distinct innate immune intracellular sensors (12, 13). Once activated, those sensors recruit the adapter molecule ASC (apoptosis-associated speck-like protein containing a caspase activating and recruitment domain, CARD) in order to activate pro-caspase-1, thus forming the inflammasome complex also known as ASC-speck (14). The enzymatic activity of caspase-1 is required for IL-1β and IL-18 processing and secretion as well as for the cleavage of the pore-forming protein gasdermin D (GSDMD), thus inducing a cell death process called pyroptosis (15, 16).

Among the distinct cytosolic sensors able to form inflammasomes, the molecule NLRP3 (NOD-, LRR- and pyrin domain-containing protein 3) has been implicated in COVID-19 (17–20). Several stress-related cellular processes have been reported to induce NLRP3 activation in response to distinct PAMPs and DAMPs, including the generation of reactive oxygen species (ROS) (21–26). Since robust redox responses observed in hypoxic conditions during other pulmonary infections are commonly associated with exacerbated cytokine production and disease progression (27–32), host oxidative stress has also been implicated in the pathogenesis of COVID-19 (33–35).

Oxidative stress results from the imbalance of total oxidant status over antioxidant response (36). Aberrant generation of ROS along with accumulation of toxic lipid peroxides can be detrimental to host cells and thus promote tissue damage. The inability of cells to detoxify biological membranes from lipid peroxidation is caused by a decrease in the enzymatic activity of glutathione peroxidase-4 (Gpx4) often due to reduced intracellular levels of its co-factor glutathione (GSH). Of note, a decrease in GSH levels has been reported in several comorbidities, namely, type-2 diabetes (37–40), obesity, cardiovascular disorders (41–43), respiratory diseases (44–47), cancer (48, 49), and hepatitis (50, 51), most of which have been recognized as risk factors for the severe outcome of COVID-19.

Therefore, we hypothesized that uncontrolled oxidative stress may form a positive feedback loop exacerbating NLRP3-inflammasome activation and the production of pro-inflammatory cytokines, such as IL-1β, during COVID-19. Here, we assessed inflammasome and oxidative stress activation in circulating blood monocytes from COVID-19 patients and investigated whether these cellular pathways cooperatively contribute to COVID-19 disease severity and cytokine release syndrome. Our findings provide insights into the pathogenesis of COVID-19, both during acute infection and early recovery, suggesting inhibition of both NLRP3 activation and lipid peroxidation as potential therapeutic interventions in COVID-19.

## Materials and Methods

### Study Participants and Approval

Study participants were enrolled in the NIH clinical protocol: COVID-19-associated Lymphopenia Pathogenesis Study in Blood (CALYPSO), NCT04401436, which recruited at the NIH clinical center, Washington Hospital Center and Georgetown University Hospital. Healthy volunteer blood samples were obtained under the protocol 99-CC-0168 and were de-identified prior to distribution. Protocols in this study were reviewed and approved by the National Institutes of Health (NIH) Central Intramural Institutional Review Board (IRB). All participants provided written informed consent prior to any study procedures in accordance with the Declaration of Helsinki. Patients enrolled in this study were classified according to their highest oxygen requirement up until the time of the research blood draw in (1) mild cases, in which patients required 4 l or less including no oxygen, (2) moderate cases, when their requirement was up to 50% oxygen concentration (FiO2), and (3) severe cases, in which patients required over 50% FiO2 or ICU care. Only patients who were able to consent themselves were eligible to participate, which limited the enrollment of critically ill ventilated ICU patients. In addition, 14 patients were sampled longitudinally, at the acute phase of the disease and after a short-term recovery period (approximately 52 days after infection onset) and 2 patients were enrolled after their recovery.

### Plasma Biomarker Measurements

Cryopreserved plasma samples from healthy control individuals (HCs) and patients were assessed to measure levels of the following inflammatory biomarkers according to the manufacturer’s instructions. Levels of IFN-γ, IL-2, IL-6, IL-7, IL-8, IL-10, IL-12p70, TNF-α, IL-27, E-Selectin, P-Selectin, ICAM-3, SAA, CRP, VCAM-1, ICAM-1 and IL-18 were quantified using electrochemiluminesence kits from Meso SCALE (Gaithersburg, MD, USA). Levels of ferritin heavy chain (ferritin-H) were determined by enzyme-linked immunosorbent assay (ELISA) from Thermo Fisher Scientific (Rockford, IL, USA). D-dimer levels were measured using Enzyme Linked Fluorescence Antibody kits from BioMerieux, MA, USA. Levels of iron were assessed using a commercial kit from Novus Biologicals (Centennial, CO). Concentrations of catalase, superoxide dismutase activity (SOD) and total oxidant status were measured using kits from Sigma-Aldrich (St. Louis, MO, USA). Levels of sCD14 and sIL-6Ra were quantified by ELISA from R&D Systems (Minneapolis, MN, USA). Lipid peroxidation in plasma was assessed using an assay kit from Cayman Chemical.

### Cell Culture and Treatments

Cryopreserved Ficoll-isolated peripheral blood mononuclear cells (PBMCs) from patients or HCs were thawed and resuspended in RPMI-1640 media (Corning, NY, USA) supplemented with 10% heat-inactivated human AB serum (Gemini Bio-Products, West Sacramento, USA) and 0.05% benzonase (MilliporeSigma, USA). Cells were rested for 1 h at 37°C and 5% CO_2_ and subsequently plated at 10^6^ cells/well in round bottom 96-well plates (Corning Costar™, MilliporeSigma, USA) followed by immune staining. Alternatively, cells were incubated for 3 h, in the presence or absence of MCC950 (3 μM; InvivoGen, CA, USA) or colchicine (10 μM; Sigma-Aldrich, St. Louis, MO, USA) for inflammasome inhibition. IL-1β, IL-6, and TNF-α were quantified in culture supernatants by using Multi-Analyte Flow Assay Kit (LEGENDplex) from Biolegend, following the manufacturer’s instructions.

### Inflammasome and Mitochondrial Status Assessment by Imaging Flow Cytometry

Inflammasome complex assembly was evaluated by detection of ASC speck formation by imaging flow cytometry, as previously described (52). Briefly, PBMCs were incubated with the fluorochrome inhibitor of caspase-1/4/5 (FAM-FLICA, Immunochemistry technologies (ICT), Bloomington, MN) following the manufacturer’s instructions, for 50 min at 37°C, to allow for binding of FLICA to activated inflammatory caspases. Cells were washed twice with the FLICA kit wash buffer and then incubated with LIVE/DEAD Fixable AQUA Dead Cells Stain (Thermo Fisher, USA) for 15 min at RT, followed by extracellular staining in PBS + 1% BSA with the following fluorochrome-conjugated antibodies for monocyte phenotyping: anti-CD14 BV605 (Clone:M5E2), anti-CD16 PE-Cy7 (Clone:3G8), and anti-CD3 PE (Clone : HIT3a) from BioLegend, San Diego, CA, USA; anti-CD20 PE (Clone:2H7), anti-CD19 PE (Clone : SJ25C1), and anti-CD2 PE (Clone : RPA-2.10), from eBioscience, San Diego, CA; anti-CD56 PE [(Clone: B159 (RUO)], anti-HLA-DR BV421 [Clone: G46-6 (RUO)], and anti-CD66b (Clone:G10F5) from BD Biosciences, Franklin Lakes, New Jersey. Cells were fixed and permeabilized with Cytofix/Cytoperm (BD Biosciences, USA) overnight at 4°C and stained for 1 h at RT for intracellular ASC, with anti-ASC/TMS1 AF647 antibody from Novus Biologicals, Littleton, CO. In parallel, cells were stained with monocyte markers and 200 nM Mitotracker Red (MitoTracker™ Red CMXRos, Invitrogen) followed by ASC intracellular staining for mitochondrial membrane potential evaluation. Cells were acquired using a 12-channel Amnis ImageStreamX Mark II (MilliporeSigma) imaging flow cytometer and the integrated software INSPIRE (MilliporeSigma) was used for data collection. Images were analyzed using image-based algorithms in the ImageStream Data Exploration and Analysis Software (IDEAS 6.2.64.0, MilliporeSigma) as described previously (52).

### Flow Cytometry

PBMCs were stained with the following list of fluorescently labeled antibodies against cell surface markers subsequently detected by flow cytometry: anti-CD14 BV605 (clone: M5E2), anti-CD16 PE-Cy7/BV711 (clone: 3G8), and anti-CD3 PE (clone: HIT3a) from BioLegend; anti-CD20 e450 (clone: 2H7), anti-CD19 e450 (clone: SJ25C1), anti-CD2 e450 (clone: RPA-2.10), and anti-IL-1β (clone: CRM56) from eBioscience; anti-CD56 e450 [clone: B159 (RUO)], anti-HLA-DR APC-Cy7 [clone: G46-6 (RUO)], and anti-CD66b e450 (clone: G10F5) and CCR2 PerCP Cy5.5 from BD Biosciences. Staining protocol proceeded as described above for imaging flow cytometry analysis. Surface Glut-1 receptor expression was detected by binding to the Glut-1 ligand fused to enhanced green fluorescent protein (GFP) (Metafora Biosystems, Evry, France), as previously described (53). In brief, 10^6^ cells were incubated for 20 min with 10 µl of Glut-1–GFP solution prior to Live/Dead staining using LIVE/DEAD Cell Viability Assay (Life Technologies, CA, USA). GFP fluorescence was detected by staining cells with Alexa Fluor 647 anti-GFP antibody at 1:200 (Biolegend). Data were acquired on a BD Fortessa flow cytometer (BD Biosciences). All compensation and gating analyses were performed using FlowJo 10.5.3 (TreeStar, Ashland, OR, USA).

### Lipid Peroxidation Assay

Lipid peroxidation in total PBMC cultures was assessed by using the Click-iT Lipid Peroxidation Imaging Kit (Life Technologies, SA, USA) according to the manufacturer’s instructions. Cells were incubated with the LAA reagent (alkyne-modified linoleic acid) for detection of lipid peroxidation-derived protein modifications at 37°C for 1 h, and then washed with 1× DPBS. After cell centrifugation (1,500 rpm for 5 min) to remove the LAA reagent, cells were incubated with antibodies to measure lipid peroxidation only in CD14^+^ monocytes. Live/Dead staining was performed as described previously above and the cells fixed by adding cytofix/cytoperm (BD bioscience, USA). After completing the LAA staining, fixed cells were washed, and resuspended in 1× DPBS, and then LAA fluorescence was analyzed by flow cytometry.

### Measurement of Intracellular GSH Levels

Intracellular GSH levels were measured in PBMC lysates by using a Glutathione Assay Kit (Cayman Chemical, USA) according to the manufacturer’s instructions. Total GSH levels were normalized by total protein concentration determined by using a Pierce BCA protein Assay kit (Thermo Fisher, IL, USA).

### Mitochondrial Superoxide Assay

Mitochondrial superoxide was detected by using a flow cytometry–based assay. Total PBMCs were washed with Hank’s balanced salt solution with calcium and magnesium (HBSS/Ca/Mg, Gibco, USA) to remove residual media by centrifugation. Cell pellet was stained with MitoSOX probe (Life Technologies, USA) at 37°C for 30 min following the manufacturer’s protocol. Cells were next washed with HBSS/Ca/Mg and incubated with antibodies to analyze CD14^+^ monocytes by flow cytometry. Cells fixed with 2% paraformaldehyde at room temperature for 30 min. Fluorescence intensity of the probe was then measured by flow cytometric analysis.

### *In Vitro* Exposure of Human Monocytes to SARS-CoV-2

Elutriated monocytes isolated from HC-derived fresh PBMCs were co-cultured with VERO E6-media containing SARS-CoV-2 USA-WA1/2020 (SARS-CoV-2) at different MOIs for 3 h at 37°C under low serum conditions (DMEM supplemented with GlutaMAX and 2% fetal bovine serum (FBS)) in a Biosafety Level 3 facility. Control cells were co-cultured with heat-inactivated VERO E6-media containing SARS-CoV-2 USA-WA1/2020 at a MOI of 1, VERO E6-media without SARS-CoV (SARS-CoV media) supernatant at a MOI of 1 or DMEM supplemented with GlutaMAX and 2% FBS media alone. After 3 h of viral exposure, monocytes were treated with Ferrostatin-1 (Fer-1) (10 µM; Selleck, USA), MCC950 (10 µM; *In vivo*gen, CA, USA) or vehicle (DMSO; Sigma-Aldrich, St. Louis, MO, USA) and then incubated for an additional 21 h at 37°C. Cell supernatants were harvested for quantification of IL-1β by ELISA following the manufacturer’s directions (Sigma-Aldrich, USA). Alternatively, cells were stained with fluorescently labeled antibodies against monocyte surface markers as described above, followed by intracellular staining with anti-IL-1β AF647 (clone: CRM56) from eBioscience, and subsequently detected by flow cytometry.

To determine viral titers by TCID50 assay, cells were harvested, lysed by freeze/thawing and plated in triplicate onto Vero E6 cells using 10-fold serial dilutions. Viral stock was used as a positive control. Plates were stained with crystal violet after 96 h to assess CPE. Viral titers were determined using the Reed–Muench method (54).

### Statistical Analyses

Statistical analyses were performed using non-parametric Mann–Whitney or Kruskal–Wallis test in GraphPad Prism 8.0.1 software (GraphPad, USA). Data are presented as median with interquartile ranges. Spearman’s correlation analyses were performed using R. Differences between groups or parameters were considered significant when *p* <0.05.

## Results

### Clinical Characteristics

In total, 47 patients were enrolled in this study during the start of the COVID-19 pandemic from March 2020 to August 2020. They were classified according to their oxygen requirement at the time of the research blood draw as outlined in methods as (1) mild (n = 31; 23 inpatients and 8 outpatients, respectively), (2) moderate (n = 4) and (3) severe (n = 12). We pooled patients displaying moderate and severe forms of the disease in the same group, which we named moderate–severe (n = 16). Two patients remained in the critical care unit and one participant died by the time the study was completed. In addition, 14 patients were followed longitudinally after a short-term recovery period (approximately 52 days after infection onset), and 2 were recruited after recovering from COVID-19 into the recovered cohort (n = 16).

The median age of the mild cohort was 63 years (IQR 50–68) with 16 (52%) women which was similar to the moderate–severe cohort that had a median age of 62.5 years (IQR 59–70) and 7 (44%) women. Time from symptom onset to sample collection was also similar between the groups [Mild: 9 days (IQR 6–13.5); Moderate–severe: 10 days (IQR 8.8–11.5)]. There was no statistically significant difference in the demographics of these two cohorts when analyzed using a Mann–Whitney test for continuous variables and a Chi-square test for categorical variables. Additional clinical and demographic features are summarized in **Table 1**. The only statistically significant differences between the two groups were a greater absolute neutrophil count (ANC) in the moderate–severe group (P <0.001) and more moderate–severe patients received remdesivir (p <0.05) which reflected clinical practice at the time.

**Table 1:** COVID-19 participant characteristics (% or median values with IQR in parenthesis).

Characteristic	Mild	Moderate-Severe	Recovered
N	31	16	16
Age—y	63 (50–67.5)	62.5 (55.8–69.5)	48.5 (37–57.5)
Female—no. (%)	16 (52)	7 (44)	12 (75)
BMI (kg/m^2^)	31 (28.6–39.5)	28.7 (24–36.4)	31.5 (25.4–48.8)
**Race—no. (%)**			
White	13 (42)	5 (31)	9 (56)
African American	14 (45)	8 (50)	3 (19)
Other	4 (13)	3 (19)	4 (25)
*ANC (×10^9^ cells/L)	3.0 (2.4–4.6)	7.3 (5.1–9.9)	3.6 (3.1–4.9)
*ALC (×10^9^ cells/L)	1.3 (1.1–2.0)	1.2 (0.9–1.8)	1.9 (1.4–2.6)
Time from symptoms to lab draw (Days)	9 (6–13.5)	10 (8.8–11.5)	52 (47.3–75.3)
**Comorbidities—no. (%)**			
Hypertension	18 (58)	12 (75)	8 (50)
Diabetes mellitus	14 (45)	6 (38)	4 (25)
COPD/Asthma	5 (16)	5 (31)	4 (25)
Renal Disease	3 (10)	2 (13)	0 (0)
**Treatment—no. (%)**			
Remdesivir	6 (19)	11 (69)	4 (25)
Dexamethasone	2 (6)	4 (25)	3 (19)

*ANC, Absolute neutrophil count; ALC, Absolute leukocyte count.

### Significant Changes in Circulating Monocyte Subsets in COVID-19 Patients

Human blood monocytes are bone marrow-derived cells that circulate in the blood for 1–3 days, and during this time undergo a process of maturation from classical CD14^high^CD16^−^ monocytes (also referred as inflammatory monocytes) into non-classical CD14^low^CD16^+^ group (also known as patrolling monocytes) through the generation of an intermediate monocyte subset (CD14^high^CD16^+^) (55, 56). These monocyte subsets exhibit very distinct functional roles in a variety of homeostatic and pathological conditions and phenotypic changes or dysregulated activation in the circulating mononuclear phagocyte compartment has been implicated in a range of inflammatory disorders, including COVID-19 (57–61). We therefore first sought to investigate phenotypic changes in circulating blood monocytes from HCs and COVID-19 patients experiencing mild or moderate-severe disease.

**Figure 1:**
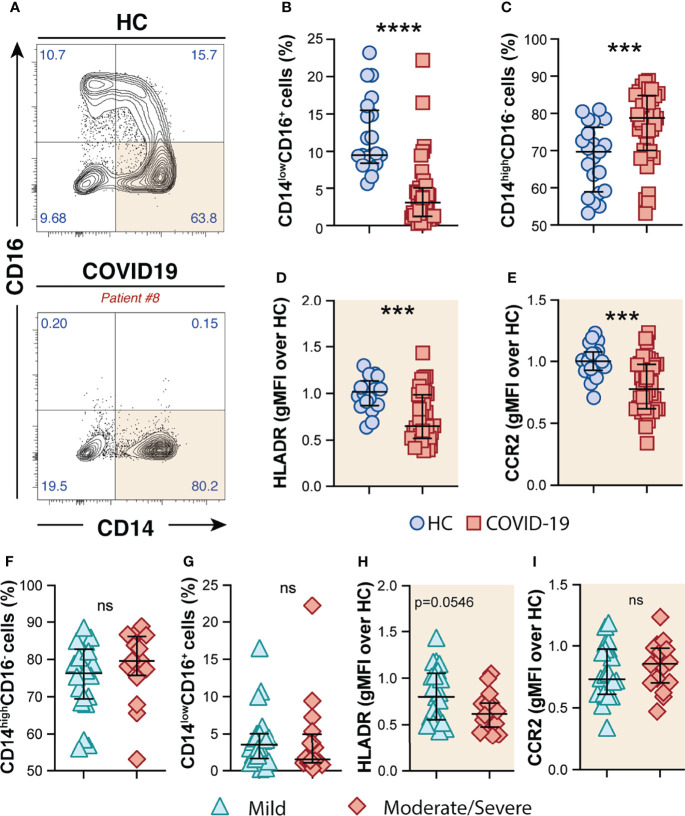
Phenotypic changes in circulating monocyte subsets during COVID-19. **(A)** Representative FACS plots showing the distribution of the monocyte subsets defined by CD14 and CD16 markers in COVID-19 patients. Percentage of CD14^low^CD16^+^ **(B)** and CD14^high^CD16^−^ **(C)** monocytes among circulating mononuclear myeloid cells (HLADR^+^CD2^−^CD3^−^CD19^−^CD20^−^CD56^−^CD66^−^) were compared between healthy controls (HC, n = 21) and COVID-19 patients (n = 38). Data are presented as median with interquartile range. The geometric mean fluorescence intensity (gMFI) of HLA-DR **(D)** and CCR2 **(E)** expression on CD14^high^CD16^−^ monocytes was compared between HC (n = 21) and COVID-19 patients (n = 38). Percentage of CD14^high^CD16^−^ **(F)** and CD14^low^CD16^+^ **(G)** monocytes and also MFI of HLA-DR **(H)** and CCR2 **(I)** expression on CD14^high^CD16^−^ monocytes is compared between COVID-19 patients experiencing mild symptoms (n = 22) versus moderate to severe forms of COVID-19 (n = 16). Data were analyzed using the Mann–Whitney test. ***P < 0.001, ****P < 0.0001; *ns*, not significant.

By analyzing the circulating monocyte compartment, we observed that COVID-19 patients displayed a striking depletion of the patrolling CD14^low^CD16^+^ subset (**Figures 1A, B**) accompanied by an enrichment of the CD14^high^CD16^−^ classical monocytes compared to HCs (**Figures 1A, C**). CD14^high^CD16^−^ monocytes were characterized by high surface expression levels of the C–C chemokine receptor type 2 (CCR2), known to be a major regulator of monocyte trafficking to inflammatory sites, while non-classical CD14^low^CD16^+^ monocytes patrol the blood vasculature, being therefore key players to survey local tissue damage (61). Interestingly, CD14^high^CD16^−^ monocytes from COVID-19 patients also showed downmodulation of CCR2 and HLA-DR molecules when compared to HCs (**Figures 1D, E**, respectively). Of note, none of these features were associated with disease severity since similar frequency of monocyte subsets and surface markers were observed when COVID-19 patients were grouped in mild and moderate–severe disease categories (**Figures 1F–I**). Collectively, these results demonstrate phenotypical and proportional changes in the peripheral monocytic compartment during SARS-CoV-2 infection, mainly characterized by shrinkage of the patrolling monocyte subset, consistent with previous reports (62–64).

### Systemic Inflammasome Activation During COVID-19 is Associated With Disease Severity

Systemic inflammation has been considered a hallmark of COVID-19 severity (7). Accordingly, several molecular measurements associated with systemic inflammation, namely, CRP, D-dimers, ferritin, and IL-6 were elevated in plasma from COVID-19 patients in our cohort (**Table 2** and **Supplementary Figure 1**). Additional analysis of these inflammatory markers failed to discriminate disease severity in this patient cohort (**Supplementary Table 1**). It is important to highlight that the COVID-19 patients were enrolled in the mild group based on their lower oxygen requirement (nasal cannula 4 l or less or no oxygen), which differs from other studies where only outpatients displaying no oxygen requirement were assigned as mild patients.

**Table 2:** Plasma biomarkers measurement in healthy controls (HC) versus COVID-19 patients (median values with IQR in parenthesis).

Biomarker (pg/ml)	HC	COVID-19	p-value
N	13	29	NA
IFN-γ	5.34 (3.2–7.8)	9.17 (4.9–24.6)	0.06
IL-10	0.36 (0.24–0.65)	0.90 (0.60–2.33)	0.003
IL-12p70	0.40 (0.22–0.76)	0.31 (0.19–1.07)	0.94
IL-2	0.43 (0.29–0.47)	0.24 (0.13–0.40)	0.21
IL-6	0.48 (0.24–0.60)	6.07 (1.06–19.1)	<0.001
IL-8	2.41 (1.53–2.89)	4.36 (2.55–6.07)	0.03
TNF-α	1.50 (1.21–1.77)	3.02 (1.67–4.73)	0.002
IL-27	1,410 (1,095–1,733)	2,295 (1,511–3,770)	0.002
CRP (mg/L)	1.81 (1.55–7.45)	83.4 (9.30–187.9)	0.005
SAA (mg/L)	7.85 (3.98–9.96)	230.8 (14.9–404.8)	<0.001
sCD14 (mg/L)	1.36 (1.23–1.66)	1.75 (1.38–2.31)	0.05
IL-6Rα	32,960 (30,321–37,174)	38,853 (34,737–44,826)	0.02
D-dimer (ng/ml)	303 (214–579)	1,452 (566–2,502)	0.002
Ferritin (ng/ml)	101 (42.7–297)	1,100 (173–1,650)	0.007

*p-value when Mann–Whitney was applied.

**Figure 2:**
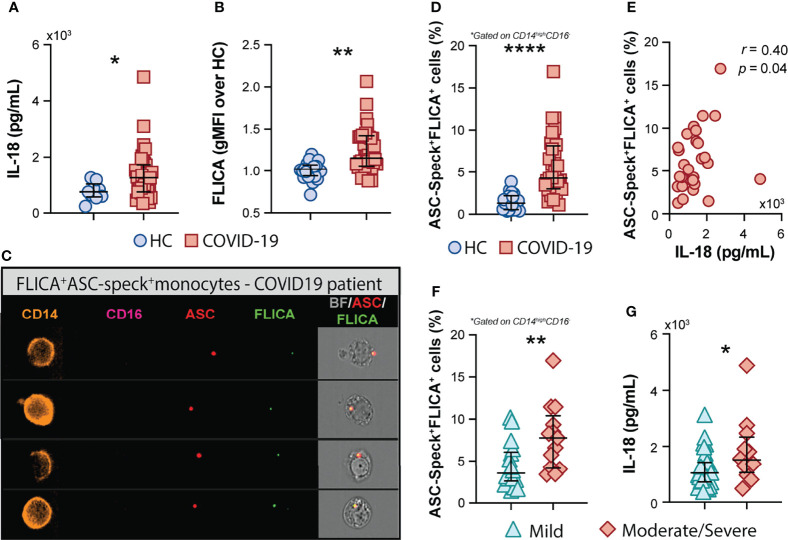
COVID-19 patients exhibit a high frequency of monocytes displaying FLICA^+^ASC-speck formation. **(A)** Plasma levels of IL-18 were compared between healthy control (HC) donors (n = 9) and COVID-19 patients (n = 39). Lines represent median values and interquartile ranges. **(B)** PBMCs were incubated with the fluorochrome inhibitor of caspase-1/4/5 (FLICA) and the geometric mean fluorescence intensity (gMFI) of FLICA within the CD14^high^CD16^−^ monocyte subset was compared between HC (n = 21) and COVID-19 patients (n = 38). Data are presented as median with interquartile range. **(C)** PBMCs from HC (n = 24) and COVID-19 patients (n = 32) were incubated with FLICA, stained for monocyte identification and intracellular ASC and acquired by Imaging flow cytometry. Representative images showing respectively: CD14, CD16, ASC and FLICA fluorescence followed by a composite image containing brightfield (BF), and the fluorescence of ASC and FLICA merged, were selected from a COVID-19 patient. **(D)** The percentage of monocytes showing spontaneous FLICA^+^ASC-Speck formation was quantified after application of Modulation_Morphology (M11,Ch11)_11-ASC feature, followed by Bright Detail Similarity R3_MC_11-ASC_2-FLICA, inside the CD14^high^CD16^−^ monocyte gate, by using IDEAS software. Lines represent median values and interquartile ranges. **(E)** Spearman correlations between plasma levels of IL-18 and the percentage of ASC-speck^+^FLICA^+^ CD14^high^CD16^−^ monocytes in COVID-19 patient samples. Percentage of ASC-speck^+^FLICA^+^ cells **(F)** and plasma levels of IL-18 **(G)** were compared between COVID-19 patients experiencing mild symptoms (n = 18 and n = 28, respectively) and moderate to severe cases of COVID-19 (n = 13 and n = 12, respectively). Lines represent median values and interquartile ranges. Data were analyzed using the Mann–Whitney test. *P < 0.05, **P < 0.01, ****P < 0.0001.

In addition to the above mentioned inflammatory markers, the inflammasome-derived cytokine IL-18 has also been implicated in COVID-19-associated pathological inflammation (9–11). Accordingly, we observed higher plasma levels of IL-18 in our cohort of COVID-19 patients when compared to HCs (**Figure 2A**), suggesting systemic inflammasome activation in this group of patients. Since drastic depletion of the patrolling monocytes subset along with increased levels of classical/inflammatory CD14^high^CD16^−^ monocytes were observed in COVID-19 patients, we evaluated whether CD14^high^CD16^−^ cells represent a source of inflammasome activation during COVID-19. To do so, peripheral blood mononuclear cells (PBMCs) from HCs and patients were incubated with FLICA, an approach widely used to measure caspase-1/4/5 activity. We observed higher caspase-1/4/5 activity within the classical monocyte subset from COVID-19 patients when compared to HCs (**Figure 2B**), suggesting this cell subset contributes to IL-1β and IL-18 maturation during COVID-19.

To directly evaluate inflammasome complex formation in CD14^high^CD16^−^ monocytes, we screened classical monocytes from HCs and COVID-19 patients for active ASC aggregates by imaging flow cytometry, following a previously described protocol (52). Briefly, PBMCs were pre-incubated with FLICA for caspase-1 detection followed by immunophenotyping and intracellular ASC staining. Using this approach, we identified monocytes with cytosolic ASC speck formation associated with active caspase-1/4/5, represented by FLICA^+^ASC-speck^+^ cells (**Figure 2C**, Merge panel). The summary data in **Figure 2D** demonstrate a substantially higher frequency of classical monocytes showing FLICA^+^ASC-speck^+^ formation in COVID-19 patients than HC subjects, which positively correlated with plasma IL-18 levels (**Figure 2E**). Importantly, in contrast to the other plasma inflammatory markers tested in this study (**Supplementary Table 1**), the percentage of monocytes with inflammasome complex formation and plasma levels of the downstream cytokine IL-18 were both significantly increased in the moderate–severe group than in patients with milder symptoms (**Figures 2F, G**, respectively), highlighting a role for inflammasomes in COVID-19 severity. Of note, inflammasome activation in circulating classical monocytes was not associated with age, sex, BMI, or race in our group of patients (**Supplementary Figure 2**).

**Figure 3:**
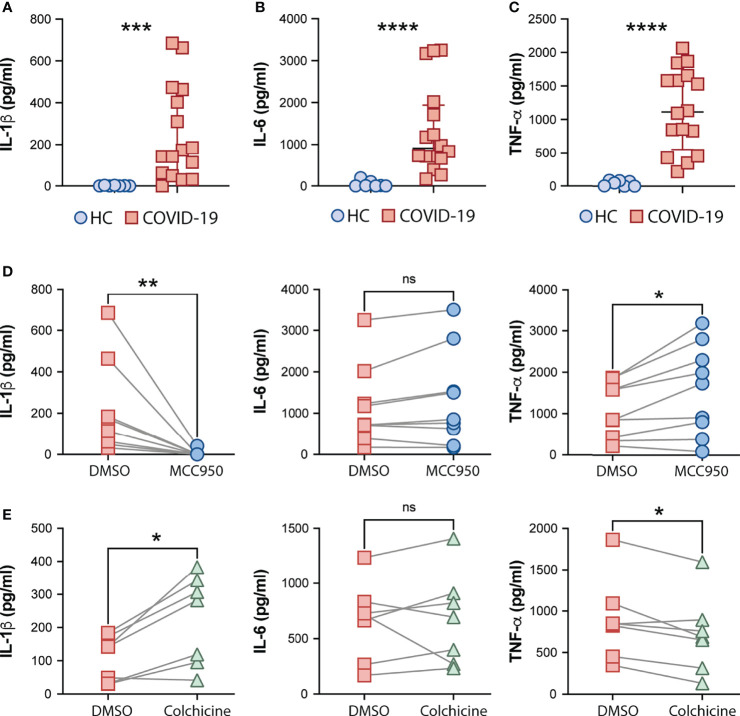
Effects of the NLRP3 inhibitor MCC950 and colchicine on cytokine secretion by COVID-19 PBMCs. IL-1β **(A)**, IL-6 **(B)** and TNFα **(C)** levels from supernatants of healthy control (HC, n = 7) or COVID-19 patients PBMCs (n = 16) after a 4-hour incubation at 37°C, were determined by multi-analyte flow assay kit. Lines represent median values and interquartile ranges. Data were analyzed using the Mann–Whitney test. ***P < 0.001, ****P < 0.0001. Alternatively, COVID-19 cells were treated with the NLRP3 inhibitor, MCC950 (10 μM; n = 9) **(D)** or colchicine (10 μM; n = 7) **(E)** during the incubation period, and cytokine levels were measured in the culture supernatants. Numbers represent the median values and interquartile ranges with or without treatment. Paired analysis was performed using the Wilcoxon test. *P < 0.05; **P < 0.01; *ns*, not significant.

Consistent with systemic inflammation, COVID-19-derived PBMCs but not HC cells spontaneously released IL-1β, and also IL-6 and TNF-α *ex vivo* (**Figures 3A–C**, respectively). We next tested the effect of MCC950, a specific inhibitor of the NLRP3 inflammasome, and colchicine, an anti-inflammatory compound, on spontaneous cytokine release from COVID-19 patient cells. In addition to other anti-inflammatory effects, colchicine has been shown to inhibit microtubule-driven spatial arrangement of mitochondria, thus preventing full NLRP3 inflammasome activation (65) and its use has been also reported to have beneficial effects in outpatients but not in hospitalized COVID-19 patients in recent clinical trials (66–68). As expected, selective inhibition of the inflammasome sensor NLRP3 (MCC950) abolished IL-1β secretion by PBMCs *ex vivo* (**Figure 3D**). Conversely, despite the promising clinical use of colchicine to prevent inflammasome activation, we observed a significant increase in the IL-1β levels released by patient-derived PBMCs *in vitro* upon colchicine treatment (**Figure 3E**). Although none of the compounds impacted IL-6 production (**Figures 3D, E** **)** by these cells, colchicine treatment reduced levels of TNF-α whereas MCC950 boosted the release of this cytokine (**Figures 3D, E** **)**.

Collectively, our findings suggest a role for NLRP3-mediated ASC-speck formation and caspase-1 activation in pathologic inflammation associated with moderate and severe forms of COVID-19 supporting potential use of NLRP3 inhibition to mitigate the exacerbated inflammasome-related responses during COVID-19.

### CD14^high^CD16^−^ Monocytes From COVID-19 Patients Exhibit Prominent Oxidative Stress Activity

**Figure 4:**
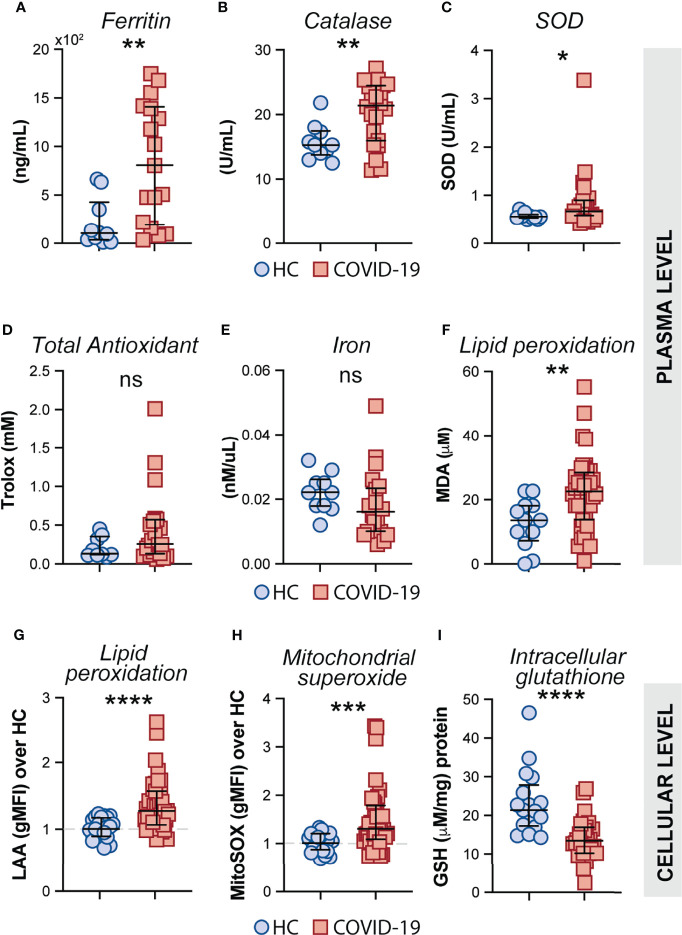
Measures of oxidative stress in COVID-19 patients. Plasma levels of ferritin **(A)**, Catalase **(B)**, Superoxide dismutase (SOD) **(C)**, Trolox **(D)** Iron **(E)** and Lipid peroxidation **(F)** were compared between healthy control (HC) donors (n = 10–12) and COVID-19 patients (n = 20–40). Lipid peroxidation (LAA) **(G)** and mitochondrial superoxide (MitoSOX) **(H)** in CD14^high^CD16^−^ monocytes was measured by flow cytometry in PBMC samples from HC (n = 22) and COVID-19 patients (n = 40). Data are expressed as the geometric mean fluorescence intensity (gMFI) over average of HC. **(I)** Intracellular glutathione (GSH) levels in PBMC lysates from HC (n = 17) and COVID-19 patients (n = 27) were determined by analytic enzymatic assay as described in *Materials and Methods*. Data are presented as median values and interquartile ranges and analyzed using Mann–Whitney test. *P < 0.05, **P < 0.01, ***P < 0.001, ****P < 0.0001; *ns*, not significant.

Oxidative stress has been suggested to be a critical host factor in driving immune response exacerbation in several infectious diseases (27–31). However, the induction of inflammatory oxidative stress responses in circulating monocytes obtained from COVID-19 patients has not been formally investigated. First, we sought to investigate the systemic oxidative response in plasma obtained from our cohort of COVID-19 patients by measuring the levels of ferritin heavy chain, catalase, total superoxide dismutase (SOD) activity, total antioxidant status, iron and lipid peroxidation (**Figures 4A–F**). Interestingly, elevated plasma levels of ferritin (**Figure 4A**), catalase (**Figure 4B**) superoxide dismutase (**Figure 4C**), and lipid peroxidation (**Figure 4F**) were found in COVID-19 patients compared to HC individuals whereas no significant difference was observed in the levels of total antioxidant response (**Figure 4D**) and iron (**Figure 4F**) in the same set of samples.

Mitochondrial superoxide is an important intracellular source of reactive oxygen species and plays a critical role in promoting Fenton/Haber-Weiss reactions by releasing iron from intracellular ferritin complex, which in turn culminates in the generation of highly reactive hydroxyl radicals (69–73). Elevated levels of hydroxy radicals in turn lead to abnormal generation and accumulation of toxic lipid peroxides affecting host cell function and viability. To carefully assess oxidative stress response in COVID-19 patients at the cellular level, we evaluated mitochondrial superoxide generation and lipid peroxidation in circulating CD14^high^CD16^−^ monocytes from COVID-19 patients (n = 40) and HCs (n = 23) by flow cytometric analysis. In addition, we used PBMC lysates from COVID-19 patients and HCs to investigate the host cellular antioxidant response by assessing intracellular levels of glutathione. We found elevated levels of mitochondrial superoxide along with aberrant lipid peroxidation in CD14^high^CD16^−^ monocytes from COVID-19 patients compared to HCs (**Figures 4G, H**, respectively). Consistent with these observations, a major reduction in intracellular glutathione levels was observed in PBMC lysates from COVID-19 patients compared to HCs (**Figure 4I**), suggesting an impaired intracellular host antioxidant response to control oxidative stress responses and ultimately lipid peroxidation.

### Exacerbated Inflammasome Activation is Associated With Mitochondrial Dysfunction in COVID-19 Patients

**Figure 5:**
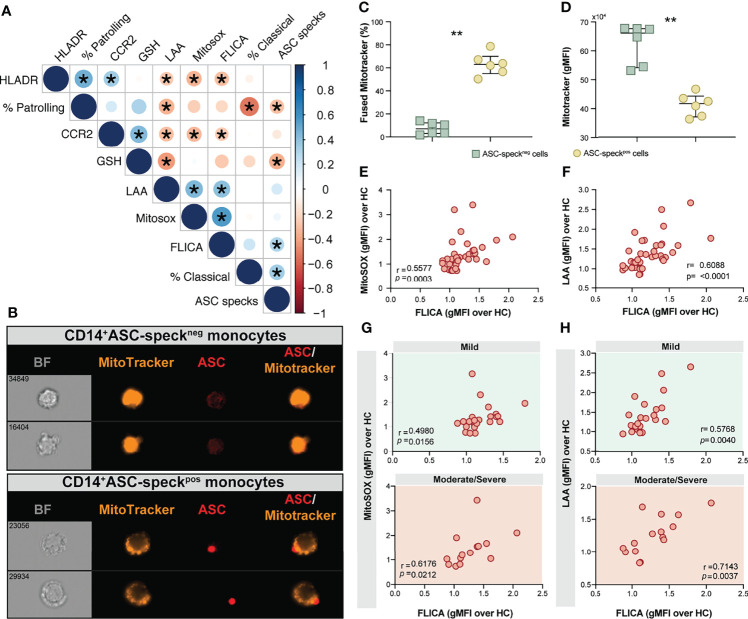
Oxidative stress and inflammasome responses are associated in COVID-19 patients. **(A)** Multi-parameter Spearman’s correlation analysis of PBMC samples from healthy control (HC) and COVID-19 patients (*P < 0.05 or less). **(B)** PBMCs from COVID-19 patients (n = 6) were incubated with Mitotracker Red, stained for intracellular ASC and acquired by means of Imaging flow cytometry. Representative images showing respectively: BF (brightfield), Mitotracker Red, ASC and a composite image of both Mitotracker Red and ASC markers merged, were selected from a CD14^+^ASC-speck^neg^ gate (top panel) and a CD14^+^ASC-speck^pos^ gate (bottom panel). **(C)** The percentage of monocytes showing the diffuse pattern for Mitotracker Red shown in **(B)** (top panel) versus the aggregated staining profile as shown in B (bottom panel) was determined after application of Major Axis Intensity_M04_Ch4 × Mean Pixel_M04_Ch4 features inside both CD14^+^ASC-speck^neg^ and CD14^+^ASC-speck^pos^ gates using IDEAS software. **(D)** Loss of mitochondrial membrane potential (ΔΨm) was determined by the change of Mitotracker Red gMFI staining on CD14^+^ASC-speck^neg^ and CD14^+^ASC-speck^pos^ subsets. Data are presented as median values and interquartile ranges and were analyzed using the Mann–Whitney test (**P < 0.01). **(E, F)** Spearman correlations between FLICA and MitoSOX **(E)** or FLICA and LAA **(F)** levels whithin CD14^high^CD16^−^ monocytes from COVID-19 patients. **(G, H)** Spearman correlations between FLICA and MitoSOX **(G)** or FLICA and LAA levels **(H)** on CD14^high^CD16^−^ monocytes obtained from COVID-19 patients grouped based on their disease severity in patients experiencing mild symptoms (top panel, green) or moderate to severe cases of COVID-19 (bottom panel, red). Each dot represents a correspondent value from each patient. *ns*, not significant.

We next sought to evaluate associations between cellular inflammatory responses and oxidative stress in monocytes from COVID-19 patients and HCs. We found strong negative correlations between lipid peroxidation, mitochondrial superoxide and inflammasome activation and the frequency of CD14^low^CD16^+^ monocytes and also CCR2 expression on CD14^high^CD16^−^ monocytes (**Figure 5A**). A highly significant negative correlation between glutathione levels and lipid peroxidation along with ASC-specks was also observed (**Figure 5A**), suggesting a possible link between cell stress responses and inflammasome activation during COVID-19.

Monocyte secretion of IL-1β and IL-18 as a result of NLRP3 inflammasome activation requires mitochondrial-derived ROS and is commonly associated with an imbalance of cellular redox status (25, 26). To assess the level of mitochondrial perturbation on monocytes isolated from COVID-19 patients, we incubated total PBMC with MitoTracker probe in order to evaluate mitochondrial membrane potential (ΔΨm) in CD14^high^CD16^−^ monocytes by imaging flow cytometry. This approach was also employed to address whether mitochondrial perturbation or dysfunction is associated with inflammasome assembly measured here by ASC-speck formation. CD14^high^CD16^−^ monocytes from COVID-19 patients were grouped according to their ASC-speck status in CD14^high^ASC-speck^neg^ (resting cells) and CD14^high^ASC-speck^pos^ cells and the loss of mitochondrial membrane potential was determined a decrease in MitoTracker MFI (**Figure 5B**). ASC-speck^pos^ monocytes showed an elevated frequency of cells displaying small puncta of MitoTracker staining compared to ASC-speck^neg^ cells (**Figure 5C**). In contrast, decreased MitoTracker gMFI was found in cells displaying ASC speck formation (**Figure 5D**), demonstrating loss of mitochondrial membrane potential and inflammasome activation in CD14^high^CD16^−^ monocytes.

Mitochondrial dysfunction is also linked with a metabolic shift from OXPHOS to glycolysis in inflammatory immune cells (74, 75). To investigate whether monocytes obtained from COVID-19 patients display this metabolic shift, we stained cells for Glut-1. Supporting our MitoTracker results, we found that CD14^high^CD16^−^ monocytes from COVID-19 patients showed elevated levels of Glut-1 (**Supplementary Figure 3A**), suggesting that these cells are undergoing intense cellular stress, reflected also by altered cell metabolism. Further, we found no difference in Glut-1 level when patients were grouped based on their disease severity status (**Supplementary Figure 3B**).

### Aberrant Oxidative Stress Response Strongly Correlates With Robust Caspase-1 Activation in COVID-19 Patients

We have shown that COVID-19 patients display elevated caspase-1 activity and accumulation of lipid peroxides in circulating CD14^high^CD16^−^ monocytes. As the oxidative stress response expressed by lipid peroxidation also modulates NLRP3-inflammasome activation and the consequent release of mature IL-1β and IL-18 (25, 26), we extended our analyses to explore the correlation between inflammasome activation and the oxidative stress response and their impact on disease severity.

We first investigated whether levels of mitochondrial superoxide in COVID-19 patients correlate with capase-1 activity. As shown in **Figure 5E** we observed a significant positive correlation between mitoSOX (gMFI) and FLICA (gMFI) in CD14^high^CD16^−^ monocytes from COVID-19 patients. In addition, we found a robust positive correlation between lipid peroxidation and active caspase-1 (**Figure 5F**). To test whether these parameters can reflect clinical disease severity, we stratified patients into mild or moderate–severe groups. We found a significant positive correlation of mitochondrial superoxide production and caspase-1 activity in monocytes from patients displaying mild and moderate/severe disease (**Figure 5G**). However, a stronger and more robust correlation was observed between lipid peroxidation and caspase-1 activity in patients in the moderate–severe group than those in the mild group (**Figure 5H**). Collectively, these findings reveal that CD14^high^CD16^−^ monocytes from COVID-19 patients display dysregulated oxidative stress responses and prominent inflammasome activation. Crosstalk between these two pathways may negatively impact the clinical course of SARS-CoV-2-infected subjects.

### IL-1β Secretion by Human Monocytes in Response to SARS-CoV-2 *In Vitro* Requires NLRP3 Inflammasome Activation and is Partially Dependent on Lipid Peroxidation

**Figure 6:**
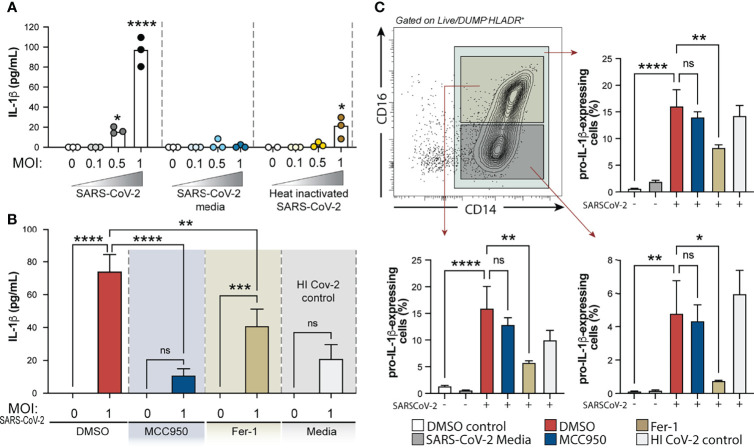
Exposure of human healthy control monocytes to SARS-CoV-2 *in vitro* induces IL-1β by a mechanism involving both inflammasome activation and lipid peroxidation. **(A)** Elutriated monocytes isolated from fresh healthy donors PBMCs were co-cultured with live SARS-CoV-2 (USA-WA1/2020) at distinct MOIs (0.1, 0.5 or 1), media only (DMSO control), VERO E6-media only (SARS-Cov-2 media), VERO E6-media containing SARS-CoV-2 (SARS-Cov-2) or heat inactivated SARS-CoV-2 (HI CoV-2 control) and IL-1β levels were measured in the culture supernatants after 24 h by ELISA. Data represent the means ± SEM of triplicate samples. Statistical significance was assessed by one-way ANOVA analysis for the indicated experimental conditions. *P < 0.05, ****P < 0.0001. **(B)** Healthy donor monocytes were incubated with SARS-CoV-2 at MOI of 1 and treated or not with NLRP3 inhibitor, MCC950 (10 μM) or lipid peroxidation inhibitor, Fer-1 (10 μM). IL-1β levels were measured in culture supernatants after 24 h by ELISA. Data represent the means ± SEM of 2 independent experiments pooled with 5–6 replicates. Statistical significance was assessed by one-way ANOVA analysis for the indicated experimental conditions. **P <0.01, ***P < 0.001, ****P < 0.0001. **(C)** Representative FACS plot showing the distribution of healthy donor monocyte subsets defined by CD14 and CD16 markers (gated on Live/Dead^low^HLADR^+^CD2^−^CD3^−^CD19^−^CD20^−^CD56^−^CD66^−^) after exposure to VERO E6-media containing SARS-CoV-2 *in vitro*. Percentage of total monocytes or CD14^high^CD16^+^ and CD14^high^CD16^−^ cells expressing pro-IL1β was compared among the distinct experimental conditions. Data represent the means ± SEM of two independent experiments in quadruplicate. Statistical significance was assessed by one-way ANOVA analysis for the indicated experimental conditions. *P < 0.05, **P < 0.01, ****P < 0.0001, *ns*, not significant.

We next sought to investigate the crosstalk between the oxidative stress pathway and NLRP3-caspase-1 activation on circulating blood monocytes during COVID-19. To do so, we incubated column-purified monocytes isolated from healthy donors with SARS-CoV-2 USA-WA1/2020 at different multiplicities of infection (MOI) and measured IL-1β in supernatants after 24 h as a result of inflammasome activation. As a negative control for active virus infection, monocytes were stimulated with heat-inactivated virus (HI-CoV-2) or VERO E6-media only (SARS-CoV-2 media). First, to evaluate the presence of replicating virus in SARS-CoV-2-exposed monocyte cultures, we incubated VERO E6 cells, a cell lineage known to be sensitive to SARS-CoV-2 infection, with SARS-CoV-2-exposed monocyte lysates serially diluted. Although we failed to detect robust SARS-CoV-2 replication within human monocytes by using this method (**Supplementary Figure 4**), SARS-CoV-2 exposure to monocytes was sufficient to induce IL-1β secretion at MOI of 1, which was not seen in cultures incubated with HI-CoV-2 at the same MOI or with SARS-CoV-2 media only (**Figure 6A**).

We then examined the direct role of NLRP3 and ROS generation on inflammasome activation in response to SARS-CoV-2 in our *in vitro* system, by incubating monocytes with NLRP3 inhibitor MCC950 or Ferrostatin-1 (Fer-1), a compound known to inhibit lipid peroxidation. Interestingly, we observed that SARS-CoV-2-induced IL-1β secretion was completely abrogated by MCC950 and partially inhibited by lipid peroxidation (**Figure 6B**).

NLRP3 activation is known to be a two-step process, where engaged TLRs or cytokine receptors give a first signal or “priming” to induce transcriptional upregulation of the NLRP3 inflammasome components (i.e. NLRP3 itself and pro-IL-1β) while a “second signal” is provided by intracellular stress pathways, thus leading to the NLRP3-ASC complex formation, caspase-1 activation and active IL-1β release. To further explore the involvement of lipid peroxidation on the cascade of NLRP3 activation in response to SARS-CoV-2, we evaluated the impact of NLRP3 and lipid peroxidation inhibition on pro-IL-1β induction by HC monocytes upon exposure to the virus *in vitro*. Of note, both drugs used in this study did not affect cell viability *in vitro* as measured by Live/Dead staining and analyzed by flow cytometry (**Supplementary Figure 5A**). Depletion of CD16^+^CD14^-^ monocytes and enrichment of CD14^high^CD16^+^ cells was observed *in vitro* upon exposure to VERO E6-media only or VERO E6-media containing SARS-CoV-2 (**Supplementary Figures 5B, C**). However, pro-IL-1β induction in these cultures was only seen when monocytes were exposed to the virus, suggesting that PAMPs from the virus are activating the pro-inflammatory response program in these monocytes. Interestingly, Fer-1-treated monocytes displayed significantly lower frequency of cells expressing pro-IL-1β which was not seen in cultures treated with MCC950 (**Figure 6C**), consistent with its effect downstream of the signal 2 for NLRP3 activation. Together, these *in vitro* results suggest that lipid peroxidation, a surrogate of oxidative stress, can further exacerbate NLRP3-inflammasome activation by amplifying the induction of pro-IL-1β in response to SARS-CoV-2.

### Excessive Oxidative Stress Response and Inflammatory Profile Persist in COVID-19 Patients After Short-Term Recovery

**Figure 7:**
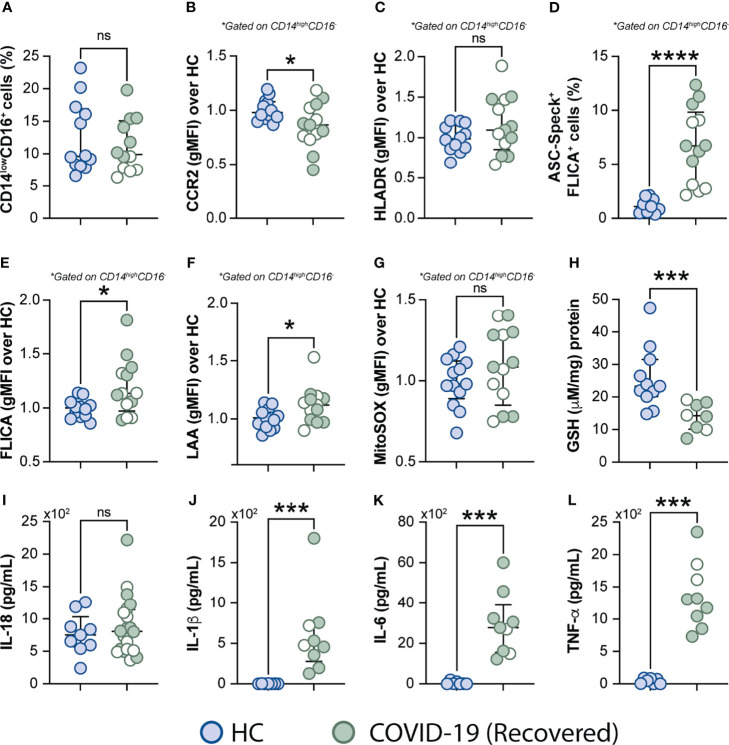
Measurements associated with inflammatory responses persist in COVID-19 patients after short-term recovery. Oxidative stress and inflammatory markers were compared between HCs and COVID-19 patients after recovery (median of 52 days IQR: 47.3–75.3, after infection onset) as follows: **(A)** Percentage of patrolling CD14^low^CD16^+^ monocyte subset, **(B)** CCR2 expression on monocytes, **(C)** HLADR expression on monocytes, **(D)** Percentage of ASC-speck^+^ CD14^high^CD16^-^ cells, **(E)** caspase-1 activity measured by FLICA staining, **(F)** lipid peroxidation (LAA) and **(G)** mitochondrial superoxide (MitoSOX) in classical monocytes, **(H)** intracellular glutathione levels in PBMC. **(I)** IL-18 levels were measured in plasma samples. **(J–L)** Spontaneous in vitro IL-1β, IL-6 and TNFα production by PBMC, respectively. Lines represent median values and interquartile ranges. Closed symbols indicate hospitalized patients (inpatients) and open symbols denote mild disease outpatients. Data were analyzed using the Mann-Whitney test. *P < 0.05, ***P < 0.01, ****P < 0.01; ns, not significant.

It has been reported that some COVID-19 patients display lingering symptoms after disease recovery. This clinical condition known as post-acute COVID-19 syndrome is not yet well understood and has been described even in patients who experienced mild disease (76, 77). Since cell stress and inflammatory measures were elevated in COVID-19 patients, we sought to investigate whether this exacerbated inflammatory status persisted after a short recovery (approximately 52 days after infection onset). To do so, we first evaluated the frequency of monocytes along with expression of phenotypic or inflammatory markers such as CCR2, HLA-DR, ASC-speck formation, caspase-1 activity, mitochondrial superoxide generation, lipid peroxidation and also cytokine production including IL-18, IL-1β, IL-6, and TNF-α (**Figures 7A–L**), by comparing those markers across recovered individuals and HCs. Among all parameters tested, we observed that CCR2 expression (**Figure 7B**), FLICA^+^ASC-speck monocyte frequency (**Figure 7D**), caspase-1 activity (**Figure 7E**), lipid peroxidation (**Figure 7F**), intracellular GSH levels (**Figure 7H**) and spontaneous cytokine release *in vitro* were all significantly altered in the COVID-19 recovered group versus HCs, indicating that short-term recovery does not afford a return to previous levels for these measurements (**Figure 7**). In contrast, longitudinal analysis revealed significant increase in CD14^low^CD16^+^ monocytes after recovery, suggesting a restoration of the normal distribution of monocytes subsets in peripheral blood (**Supplementary Figure 4A**). Additionally, measurements associated with metabolic shift (**Supplementary Figure 3C**), cell activation, inflammasome, oxidative stress, as well as pro-inflammatory cytokine production showed no significant difference between the acute and early recovery phase (**Supplementary Figures 6B–L**). Importantly, the sustained cellular stress and inflammatory status found in the COVID-19 patients post-recovery was not associated with initial disease severity, since this inflammatory profile was observed regardless of patient classification at the acute phase of the disease as inpatients (closed symbols) or outpatients (open symbols) (**Figure 7** and **Supplementary Figure 6**). These findings suggest that COVID-19-related stress and inflammatory responses persist after short-term recovery even in patients displaying mild symptoms at the acute phase of disease.

## Discussion

In SARS-CoV-2 infection, inflammatory monocyte-derived macrophages are the prominent immune cells in the infected lungs, playing a pivotal role as a major source of inflammatory mediators and thus significantly contributing to disease progression (78, 79). Herein, we expand this knowledge by showing that approximately 10% of circulating CD14^high^CD16^−^ inflammatory monocytes from COVID-19 patients demonstrate ASC/Caspase-1/4/5 inflammasome complex formation, which appears to persist after short-term recovery. These aggregates are required for caspase-1-mediated IL-1 and IL-18 secretion, highlighting the role of CD14^high^CD16^−^ monocytes in COVID-19-related inflammation. Enhanced caspase-1 activity on these cells strongly correlated with dysregulated oxidative stress status evidenced by mitochondrial dysfunction, mitochondrial superoxide generation and lipid peroxidation. Such clinical findings were confirmed *in vitro*, with oxidative stress identified to be partially involved in NLRP3-mediated IL-1β secretion by monocyte cultures exposed to SARS-CoV-2. Importantly, elevated inflammasome activation and aberrant oxidative stress response were both strongly correlated with worse clinical disease outcome, supporting the concept that myeloid-derived inflammatory responses are closely associated with disease severity in COVID-19.

Marked phenotypic differences were observed in the monocytic compartment in COVID-19 patients, namely, a striking shift towards the CD14^high^CD16^−^ classical/inflammatory monocyte subset and downmodulation of HLA-DR and CCR2 markers in those cells as reported in other studies (62, 64). More recently, the SARS-CoV-2 protein ORF7a has been shown to interact with CD14^+^ monocytes, triggering downmodulation of HLA-DR and production of proinflammatory cytokines *in vitro* (63). It is not clear, however, whether specific viral factors may also contribute to the downregulation of CCR2 on classical monocytes. CCR2 expression on these cells is known to mediate bone marrow egress and recruitment into inflamed tissues in response to CCL2 (80) which seems to be elevated in serum and bronchoalveolar fluid (BALF) samples obtained from COVID-19 patients (60, 62, 81). Thus, our data suggest downmodulation of CCR2 as a possible compensatory mechanism in response to sustained overproduction of CCL2 or continuous trafficking of CCR2^+^ cell into tissues (82).

Furthermore, massive migration of classical monocytes to inflamed tissues could prevent these cells to differentiate into the non-classical monocytes, which may partially explain their disappearance from the peripheral blood observed in COVID-19 and many other inflammatory diseases. In our *in vitro* experiments with the virus, however, we have also observed elevated inflammasome-mediated responses mainly within the inflammatory and intermediate monocyte subsets, with profound reduction in frequencies of patrolling cells. Although it is well known that the patrolling cells do not survive long cultures and classical monocytes undergo differentiation towards the intermediate phenotype after stimulation *in vitro* (83), taken together, our findings could also indicate that CD14^high^ monocytes are more prone to interact with viral proteins such as SARS-CoV-2 ORF7a, thus triggering inflammasome activation and cytokine production (63). Consistent with this hypothesis, one study demonstrated ACE2 mRNA and protein expression levels are higher in classical monocytes when compared to the other two subsets (84), which may facilitate interactions with SARS-CoV-2. Further investigations are needed to elucidate whether distinct monocyte subsets present intrinsically different responses to SARS-CoV-2-mediated inflammasome activation.

Furthermore, while some reports have shown that SARS-CoV-2 can actively infect primary human monocytes, resulting in NLRP3 activation and IL-1β secretion (20, 85, 86), in the present study we were unable to detect productive infection of primary human HC monocytes by SARS-CoV-2 *in vitro* (**Supplementary Figure 4**), although SARS-CoV-2 exposure was sufficient to promote NLRP3-driven IL-1β production. A possible explanation for the discrepancy between our results and others relates to the different approaches used to evaluate SARS-CoV-2-monocyte infection. In this regard, here we evaluated productive SARS-CoV-2 infection by exposing VERO E6 indicator cells which are known to be virus susceptible, to monocyte lysates, whereas other studies have employed rt-PCR or fluorescent-protein reporter virus to evaluate SARS-CoV-2 infection in human monocyte cultures. Regardless, our observation is in agreement with other recent reports showing that monocytes, monocyte-derived macrophages and dendritic cells can be activated by SARS-CoV-2 to induce cytokine production even in the absence of productive infection (64, 87). Moreover, it was also recently shown that distinct SARS-CoV-1/2-derived glycoproteins were able to induce NLRP3 inflammasome activation in THP-1 macrophages, thus suggesting interactions of these proteins with monocytes are sufficient to promote inflammasome activation independent of active infection (88). These findings, however, do not rule out the possibility that monocytes/macrophages are able to be productively infected in patients. In addition to ACE2 binding, other potential routes of SARS-CoV-2 entry in monocytes have also been proposed such as binding of the spike protein to the CD147 receptor, antibody-mediated phagocytosis *via* Fc gamma receptors, as well as engulfment of dying infected cells (86, 89, 90). Nonetheless, further studies with SARS-CoV-2 variants displaying different mutations are also needed to investigate whether changes in the viral genome may affect viral uptake by monocytes/macrophages.

Nevertheless, our results indicate NLRP3 as the main inflammasome sensor promoting the poor outcome of COVID-19, in agreement with other reports (20, 85, 86, 91). In fact, the presence of NLRP3 inflammasome aggregates has been previously described in PBMCs and autopsy tissue samples from COVID-19 patients (20, 92). Our findings extend these observations by revealing that aberrant oxidative stress responses evidenced by mitochondrial superoxide production and lipid peroxidation strongly associate with caspase-1 activity, suggesting a crosstalk between oxidative stress and inflammasome activation in SARS-CoV-2 infection. In fact, intracellular ROS production has been suggested as a key trigger of NLRP3 inflammasome activation as a result of either direct pathogen sensing or in response to DAMPs released from dying cells (25, 26). Previous studies have reported that SARS-CoV transmembrane pore-forming protein, known as viroporin SARS-CoV 3a or ORF3a, activates the NLRP3 inflammasome complex in LPS-primed macrophages through a mechanism dependent on K+ efflux and mitochondrial ROS (93). In addition, ORF3a was also shown to promote inflammasome assembly through TRAF3-mediated ubiquitination of ASC, while another viral protein, ORF8b seems to interact directly with the leucine-rich repeat domain of NLRP3 to stimulate its activation independent of ion channel activity (94). More recently, an *in vitro* study showed that SARS-CoV-2 viroporin protein induces the NLRP3-inflammasome formation in a lung epithelial cell line (95), suggesting that SARS-CoV-2 may trigger ROS-mediated NLRP3-inflammasome activation similarly to SARS-CoV. In this regard, we demonstrated that lipid peroxidation triggered by SARS-CoV-2 exposure amplifies the induction of pro-IL1β in HC monocyte cultures, and thus exacerbates the release of the mature form of this pro-inflammatory cytokine.

Our data also support the use of specific NLRP3 inhibitors, such as MCC950, to attenuate inflammasome-related responses during COVID-19, since this drug was able to abrogate *ex-vivo* IL-1β secretion, conversely to colchicine, which decreased TNF-α release. Accordingly, the ability of colchicine to bind to free tubulin dimers, thus blocking microtubule polymerization and therefore TNF-α secretion, is believed to be the key mechanism behind the broad spectrum of colchicine-mediated anti-inflammatory effects (96, 97). On the other hand, MCC950 specifically binds to NLRP3, thus inhibiting its activation (and thereby caspase-1-dependent active IL-1β secretion), without affecting pro-IL-1β or other cytokine production downstream of TLR engagement, consistent with our observations. The upregulation of IL-1β and TNF-α respectively by colchicine and MCC950, however, was a bit surprising, and could possibly be explained by the interplay between these two cytokines in self-regulating each other, although further studies must be conducted to clarify this issue.

A major observation of the present study is that the inflammatory profile of circulating monocytes persists in COVID-19 patients, post-recovery regardless of their disease severity during acute infection (**Figure 7** and **Supplementary Figure 6**). It has been reported that about 80% of hospitalized patients with COVID-19 displayed at least one long-term symptom for several months after discharge, particularly fatigue and dyspnea (98–100). Moreover, lingering symptoms and functional impairment up to eight months after contracting mild SARS-CoV-2 infection was also reported in a group of low-risk healthcare workers (101). Indeed, some patients in our cohort, who had experienced either mild or severe symptoms during acute COVID-19, have also reported the persistence of at least one of the current known COVID-19 symptoms following recovery from infection. However, we were unable to establish a close association between the biomarkers tested in this study and any of the residual symptoms post-recovery. The pathophysiology, risk factors and treatment of post-acute COVID-19 is currently poorly understood. In that sense, our observations of sustained dysregulated oxidative stress and inflammasome activation in monocytes after short-term recovery support one of the current hypothesis that long term COVID-19 is driven by persistent pathological inflammation and suggest the pathways involved as potential targets for therapeutic intervention (76, 77). In this regard, it will be important to evaluate whether persistent immune activation after COVID-19 recovery relies on the presence of undetectable SARS-CoV-2 virus, viral RNA and/or viral antigens and also assess the contribution of unrepairable cellular and tissue damage.

The limitations of the current study include the lack of critically ill ventilated ICU patients (as participants had to be able to provide consent), the lack of multiple longitudinal sampling and small sample size. Nevertheless, our findings collectively corroborate the hypothesis that early exposure of alveolar macrophages and monocytes to SARS-CoV-2 may induce oxidative stress dysregulation and aberrant cytokine secretion. This initial cell priming event may contribute to the enhanced recruitment of pre-activated circulating monocytes committed to inflammasome activation and aberrant oxidative stress response to the site of infection, resulting in exacerbation of tissue immunopathology. In this context, recent advances in targeting ROS by administration of glutathione or its precursors (N-acetyl-cysteine, NAC), and also IL-1 signaling by administrating Anakinra, a IL-1 Receptor antagonist (IL-1Ra), have been reported to prevent inflammation and/or worse clinical outcome in COVID-19 patients (102–108). Thus, our findings suggest that early treatment with antioxidants and IL-1 signaling inhibitors may represent potential therapeutic intervention for COVID-19, and that such interventions may also have a role in the recovery phase of the disease.

## Data Availability Statement

The original contributions presented in the study are included in the article/**Supplementary Material**. Further inquiries can be directed to the corresponding authors.

## Ethics Statement

Protocols in this study were reviewed and approved by the National Institutes of Health (NIH) Central Intramural Institutional Review Board (IRB). The patients/participants provided their written informed consent to participate in this study.

## Author Contributions

SL, EA, AS, and IS were involved in the conception and design of the study and experiments. SL, EA, KH, AR, SN, and JR performed experiments and/or data analysis. EL, PK, GW, RP, FG, AK, JR, MM, AL, and IS recruited patients, assisted in clinical care and helped with data curation and/or visualization. AR, AS, IS, JPS, and HH provided resources and reagents. SL, EA, and IS were involved in drafting the manuscript. All authors contributed to the article and approved the submitted version.

## Funding

This project was supported in part by the intramural research program of NIAID/NIH, with federal funds from the National Cancer Institute, National Institutes of Health, under the following Contract Numbers. (HHSN261200800001E, HHSN2612015000031 or 75N910D00024). KH was also supported by a Malaghan Institute Postdoctoral Fellowship. The content of this publication does not necessarily reflect the views or policies of the Department of Health and Human Services, nor does mention of trade names, commercial products, or organizations imply endorsement by the U.S. Government.

## Conflict of Interest

Authors AR and AK were employed by company Leidos Biomedical Research, Inc.

The remaining authors declare that the research was conducted in the absence of any commercial or financial relationships that could be construed as a potential conflict of interest.

## Publisher’s Note

All claims expressed in this article are solely those of the authors and do not necessarily represent those of their affiliated organizations, or those of the publisher, the editors and the reviewers. Any product that may be evaluated in this article, or claim that may be made by its manufacturer, is not guaranteed or endorsed by the publisher.
